# The intensity of physical activity influences bone mineral accrual in childhood: the childhood health, activity and motor performance school (the CHAMPS) study, Denmark

**DOI:** 10.1186/1471-2431-13-32

**Published:** 2013-03-02

**Authors:** Malene Heidemann, Christian Mølgaard, Steffen Husby, Anders J Schou, Heidi Klakk, Niels Chr Møller, René Holst, Niels Wedderkopp

**Affiliations:** 1 Hans Christian Andersen Children’s Hospital, Odense University Hospital, Sdr. Boulevard 29, Odense C DK-5000, Denmark; 2 Spine Centre of Southern Denmark, Hospital Lillebaelt, Middelfart, Denmark; 3 RICH, Centre of Research in Childhood Health, University of Southern Denmark, Odense, Denmark; 4 Institute of Regional Health Research, Department of Biostatistics, University of Southern Denmark, Odense, Denmark; 5 Department of Nutrition, Exercise and sport, Faculty of Science, University of Copenhagen, Copenhagen, Denmark

**Keywords:** Dual energy X- ray absorptiometry, Bone health, Physical activity, Accelerometers

## Abstract

**Background:**

Studies indicate genetic and lifestyle factors can contribute to optimal bone development. In particular, the intensity level of physical activity may have an impact on bone health. This study aims to assess the relationship between physical activity at different intensities and Bone Mineral Content (BMC), Bone Mineral Density (BMD) and Bone Area (BA) accretion.

**Methods:**

This longitudinal study is a part of The CHAMPS study-DK. Whole-body DXA scans were performed at baseline and after two years follows up. BMC, BMD, and BA were measured. The total body less head (TBLH) values were used. Physical activity (PA) was recorded by accelerometers (ActiGraph, model GT3X). Percentages of different PA intensity levels were calculated and log odds of two intensity levels of activity relative to the third level were calculated. Multilevel regression analyses were used to assess the relationship between the categories of physical activity and bone traits.

**Results:**

Of 800 invited children, 742 (93%) accepted to participate. Of these, 682/742 (92%) participated at follow up. Complete datasets were obtained in 602/742 (81%) children. Mean (range) of age was 11.5 years (9.7-13.9). PA at different intensity levels was for boys and girls respectively, sedentary 62% and 64%, low 29% for both genders and moderate to high 9% and 7% of the total time. Mean (range) BMC, BMD, and BA was 1179 g (563–2326), 0.84 g/cm^2^ (0.64-1.15) and 1393 cm^2^ (851–2164), respectively. Valid accelerometer data were obtained for a mean of 6.1 days, 13 hours per day.

**Conclusions:**

There 7was a positive relationship between the log odds of moderate to high-level PA versus low level activity and BMC, BMD and BA. Children with an increased proportion of time in moderate to high-level activity as opposed to sedentary and low-level activity achieved positive effects on BMC, BMD and BA.

## Background

Osteoporosis is a highly prevalent disease [1,2], which is costly for society [3]. The disease is characterized by systemic impairment of bone mass, bone strength and alterations in the bone micro architecture resulting in an increased risk of fractures [4]. Research has focused on the treatment of osteoporosis and the consequences of the disease. However, it is equally important to focus on disease prevention during childhood and adolescence. Peak bone mass (PBM) is the highest bone mass an individual obtains during a lifetime [5]. Factors that determine PBM are not fully understood. Studies indicate that both genetic factors and lifestyle, such as diet and physical activity during childhood can contribute to optimal bone development [6]. Low PBM is an important determinant of later osteoporosis and risk of fractures [7].

Previous research has shown positive effects of weight-bearing exercise on bone mineral accrual [8] and PA undertaken in childhood have sustained lasting positive influence on the adult skeleton [9].

Dual Energy X-ray Absorptiometry (DXA) can evaluate bone health by providing estimates of bone mineral density (BMD), bone mineral content (BMC), and bone area (BA). Workload during physical activity can be measured by oxygen consumption (VO_2_) and heart rate (HR) [10]. These measures correspond well to an individual’s speed or power output [10]. However, neither of these methods were well suited for large-scale population based studies. The recently developed ActiGraph GT3X monitors use a triaxial accelerometer and provide activity counts for each vector as well as a composite vector magnitude of the three axes [11]. This method gives the opportunity to capture the complexity of habitual activity and to stratify the activity intensity into sedentary, low, moderate, and high activity by using the data from the vertical axis. The results from the vertical axis were used in this research study.

It still remains uncertain to what degree physical activity (PA) as measured by accelerometers has an impact on children’s bones, and there is no knowledge about which level, intensity or volume of PA is the most beneficial regarding bone mineral accrual in childhood.

Previous studies have reported cross-sectional data on the relationship between PA and bone health in childhood [12,13], but the longitudinal data on this relationship has not been published. This study provides such longitudinal data. The aim was to evaluate how habitual PA, defined as any PA involving muscle force including walking, running, cycling as well as more passive activities, influences bone health. The specific objective was to assess the relationship between physical activity at different intensities, measured by accelerometers, and BMC, BMD and BA accrual measured by DXA scans during a two-year period.

## Methods

### Study design

The study is a sub study of the CHAMPS study, DK, a natural experiment [14]. The study embraces ten public schools in the municipality of Svendborg, Denmark, with children from the pre-school year to 4th grade. Six schools chose to implement four additional lessons of physical education (PE) to their usual PE program and to educate PE-teachers in special age- related training principles (“sports schools”), and were matched to four schools continued with two PE lessons as usual (“normal schools”) based on school size and location. The study has been described in details elsewhere [14].

A subsample comprised of children attending 2nd to 4th grade (7.2-12 years) at baseline in year 2008 was formed for this study. These children were invited to participate in DXA scans. The children were examined by DXA at baseline and the follow-up examination was performed after two years. Accelerometer measurements were performed in the middle of the two-year test period. Examinations of the children took place at The Hans Christian Andersen Children’s Hospital, Odense, Denmark.

### Ethics

Participation was voluntary. Children and parents received information about the study through school meetings and written information. The parents signed informed consent forms. Permission to conduct The CHAMPS Study–DK was granted by the Regional Scientific Ethics committee (Project number: S-20080047).

### Data collection

#### Anthropometrical data

Body weight was measured to the nearest 0.1 kg on an electronic scale, SECA 861. Height was measured to the nearest 0.5 cm using a portable stadiometer, SECA 214 (both Seca Corporation, Hanover, MD). Body weight was measured in a thin T-shirt and stockings and both anthropometric measures were conducted barefoot.

#### Dual energy X ray absorptiometry

Dual Energy X ray Absorptiometry (DXA), GE Lunar Prodigy (GE Medical Systems, Madison, WI), ENCORE software (version 12.3, Prodigy; Lunar Corp, Madison, WI), measured BMC, BMD and BA. The total body less head (TBLH) values was used. The children were instructed to lie still in a supine position wearing underwear; a thin T-shirt, stockings and a blanket for the duration of the DXA scan. The typical scan duration was 5 min, depending on subjects’ height and weight. The instrument automatically altered scan depth depending on the size of the subject, as estimated from age, height, and weight. Two operators performed the DXA scans. The data form the DXA scans were analyzed by one person (MH). The DXA scanner was reset every day following standardized procedures. The GE Lunar Prodigy has reproducibility with precision errors (1 SD) of approximately 0.75% CV (Coefficient of Variation) for bone mass in children and adolescents with a mean age 11.4 years (5–17 years) [15].

#### Pubertal self- assessment

The children were presented with standard pictures showing the pubertal Tanner staging [16] and asked to indicate which stage best referred to their own pubertal stage. The Tanner pubertal stages self-assessment questionnaire (SAQ) used in this study consists of drawings of the 5 Tanner stages for pubic hair and breast development, respectively [17]. Explanatory text in Danish supported the self-assessment. Boys were presented with pictures and text of Tanner staging for pubic hair development, whereas girls were presented with pictures and text representing breast development and pubic hair. The procedure took place in a private space with sufficient time to self assess the pubertal stage.

#### Physical activity

Assessments were performed in the middle of the two-year test period (November 2009 to January 2010), when the children attended 3rd- 5th grade. Physical activity was assessed using the Actigraph GT3X accelerometer. The GT3X is a light, solid-state triaxial accelerometer, designed to monitor human activity and provide an estimate of energy expenditure. It measures the rate of acceleration in the (Cartesian coordinate system) z-axis/medio-lateral axis, x-axis/anterior-posterior axis and the y-axis/vertical axis. In the Actigraph GT3X, the signal is digitalized and passed through a filter with band limits of 0.25-2.5 Hz in order to help eliminate extraneous accelerations not due to human movement (e.g., vibration). The measurements of the vertical axis were used in this study [18]. The accelerometer was set to accumulate PA data every 2 seconds (2-sec. epoch) and subsequently collapsed to 10 seconds epoch [19].

Verbal and written information and instructions were given to the children along with their parents. The children were instructed to wear the device from the time they woke up in the morning until bedtime in order to capture their entire physical activity (PA) for each day, for 7 full consecutive days, thus theoretically including all weekdays and a full weekend. The children should remove the monitor when showering or swimming in order to prevent damage to the device. After the measurement period, the accelerometers were recollected and data downloaded to a computer.

#### Data reduction and analysis

The customized computer program, Propero [20], was used to process accelerometer data files. Propero was set up to include only activity in different time blocks depending on grades (2nd grade: 07.00-20.30 hours, 3rd grade: 07.00-21.00 and hours, 4th grade: 07.00-21.00 hours) to avoid measurements during sleeping hours as some participants forgot to take off the accelerometer during the night. Furthermore, in order to distinguish between true intervals of inactivity and “false intervals” of inactivity recorded when the monitor had been taken off, all strings of consecutive zero for 20 min or more were defined as “accelerometer not worn” and subsequently deleted from the summation of activity. Thus, these periods did not contribute to the required minimum of valid registered activity.

Activity data were included for further analyses if the child had a minimum of 4 separate days with 10 hours per day of valid recording after the removal of non-wear time.

Cut-off points for activity intensity levels were defined according to Evenson *et al.*[18] (see Table 1).

**Table 1:** **Classification of physical activity intensity based on Evenson accelerometer cut-off points and MET thresholds**[18]

**Physical activity intensity**	**Accelerometer cut points**	**Units of metabolic equivalent (MET)**
Sedentary activity	≤ 100 counts/min	METs < 1.5
Light physical activity	> 100 counts/min	1.5 ≤ METs < 4
Moderate physical activity	≥ 2296 counts/min	4 ≤ METs < 6
Vigorous physical activity	≥ 4012 counts/min	METs ≥ 6

### Statistical analysis

Descriptive statistics were presented as means and SD, and medians and lower and upper quartiles. Explorative plots assessed linearity between the outcome variables (BMC, BMD and BA) and the covariates. Non-linear covariates were transformed to achieve linearity. Shapiro-Wilk’s test and q-q plots were used to check assumptions of normality. Residuals plots were inspected to check for variance of homogeneity. These tests did not indicate any violations of the model assumptions.

A multilevel linear regression model (using the xtmixed option from STATA 12.1), taking into account the hierarchical structure of the data was used to assess the relationship between BMC, BMD and BA accretion and the categories of physical activity intensity levels. Models were checked by residual plots. Effects with p-values < 0.05 were considered significant.

Backward elimination was used for reduction from an initial model, containing all the explanatory variables that included height, log(weight), age and puberty at follow up, bone outcome at baseline and interactions between gender and the activity levels as well as interactions between gender and puberty. BMC was in the final model adjusted for BMC at baseline, BA and height at follow up to avoid the possibility of size- related artifacts in the analysis of bone mineral data [21], and puberty at follow up and gender. BMD was in the final model adjusted for BMD at baseline log(weight); height and puberty at follow up. BA was adjusted for BA at baseline, log(weight), height, age and puberty at follow up and gender, all representing fixed effects. School and class were chosen as random effects. The information in the regression analyses was weighed by the total days of accelerometer measurements accepted (using the pweight option from STATA 12.1). By adjusting for baseline DXA outcomes, we captured the bone accrual during the two-year follow up period.

The accelerometer output generated the child’s number of minutes, in each activity intensity level per day. The data were further converted into proportions π_*s*_*,* π_*l*_ and π_*mh*_ of activity in “sedentary”, “low” and “moderate to high” intensity intervals, respectively.

The inherent ties in the proportions π_*s*_*,* π_*l*_ and π_*mh*_ (adding up to 100%) were handled by choosing low activity level as a reference level and calculating the log odds of the proportions of the two remaining intensity levels relative to the proportion of the reference level.

**Figure 1:**
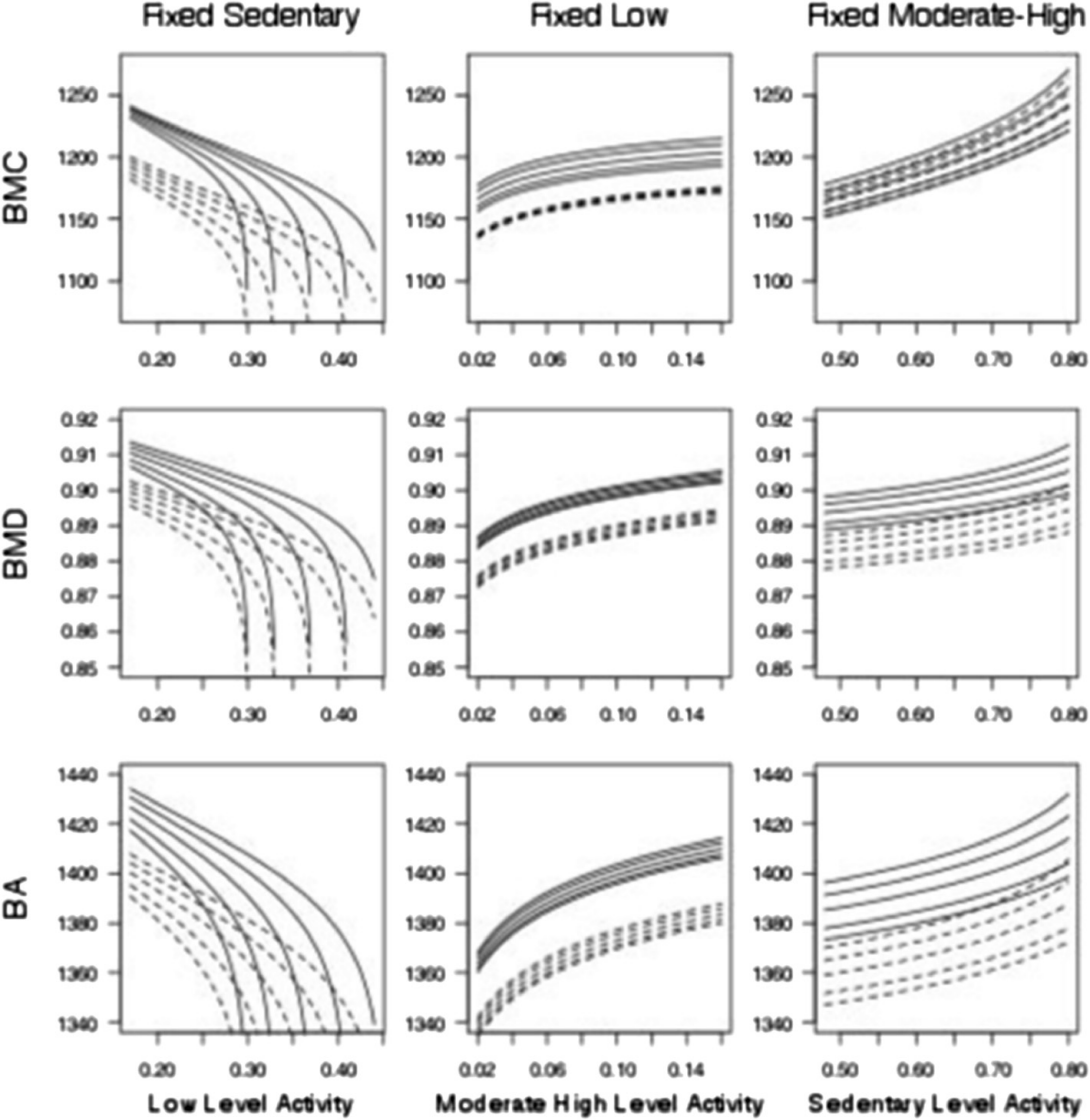
Graphs presenting the effect of changes in different configurations of the proportion of total time in physical activity and sedentary, low and moderate to high level activity on the bone traits BMC, BMD and BA.

This way the physical activity was represented by the two parameters $\theta_{\mathrm{mh}}\;=\;\text{log}\;\left(\frac{\pi_{\mathrm{mh}}}{\pi_{l}}\right)$ and $\theta_{s}\;=\;\text{log}\;\left(\frac{\pi_{s}}{\pi_{l}}\right)$. These parameters cannot be interpreted separately but must be understood as an entity. This difficulty is addressed by visualizing how the bone health measures depend on PA (Figure 1). The effect upon the outcome variables of a change in activity intensity levels depends on the relative change among the proportions and is therefore suitably assessed for changes in particular configurations. This is demonstrated by an example in the result section.

## Results

A total of 1512 children from the preschool year to 4th grade (age range 5.5-11 years) were invited to participate in The CHAMPS study-DK from baseline in September 2008, of which 1210 (80%) accepted.

A sub group was created for the present study. This group comprised of children from 2nd to 4th grade (7.2-12 years) at baseline. Of these, 742/800 (93%) accepted the invitation to participate and 682/742 (92%) participated at two-year follow up (49% boys, 51% girls). Complete datasets were obtained in 602/742 (81%) children. The characteristics of the participants at follow up regarding age, gender, anthropometry, densitometry, accelerometer data are reported in Table 2. Children, n = 152 (97 girls, 55 boys) reported Tanner stage 1, n = 263 (124 girls, 139 boys) Tanner stage 2, n = 189 (103 girls, 87 boys) Tanner stage 3, n = 38 (23 girls, 15 boys) Tanner stage 4 and n = 5 (2 girls, 3 boys) Tanner stage 5.

**Table 2:** Descriptive statistics of the participants with complete datasets n = 602 at follow up

**Variable**	**Sex**	**Mean (SD)**	**25th%**	**Median**	**75th%**
Age (yrs.)	Boys	11.5 (0.89)	10.8	11.4	12.2
	Girls	11.5 (0.87)	10.7	11.4	12.2
Height (cm)	Boys	151.2 (8.88)	145	151.1	156.5
	Girls	150.5 (8.3)	144.4	150.4	156.5
Weight (kg)	Boys	40.7 (8.46)	34.5	39.3	45.2
	Girls	40.9 (8.81)	34.8	40.05	46
Fat mass (kg)	Boys	8.2 (5.02)	4.6	6.8	10.2
	Girls	10.5 (5.0)	6.8	9.5	13.03
Lean mass (kg)	Boys	30.7 (4.97)	27.2	30.1	33.2
	Girls	28.4 (4.96)	24.5	27.8	31.8
BMC (g)	Boys	1189 (267)	1007	1169	1314
	Girls	1183 (313)	945.5	1142	1363
BMD (g/cm^2^)	Boys	0.83 (0.06)	0.79	0.83	0.87
	Girls	0.83 (0.08)	0.78	0.83	0.88
BA (cm^2^)	Boys	1405 (222)	1246	1399	1540
	Girls	1390 (249)	1211.2	1372.3	1545.5
Accelerometer (Days registered)	Boys	6.1 (0.97)	5	6	7
	Girls	6.1 (0.96)	5	6	7
Sedentary Activity (%)	Boys	62	58	62	65
	Girls	64	61	64	68
Low activity (%)	Boys	29	26	29	31
	Girls	29	26	29	31
Moderate to high activity (%)	Boys	9	6	7	9
	Girls	7	6	7	9
Pubertal stages	Boys (n)	Girls (n)			
Tanner stage 1 (n)	55	97			
Tanner stage 2 (n)	139	124			
Tanner stage 3 (n)	87	102			
Tanner stage 4 (n)	15	23			
Tanner stage 5 (n)	3	2			
Fishers exact test			<0.001*		

October 2010 to March 2011. Figures are means (SD) presented along with median, 25th% and 75th%.

Note: *The Fishers exact refers to the comparison of the pubertal stages between boys and girls at follow-up.

The relationship between BMC and the PA intensity levels represented by *θ*_*mh*_ and *θ*_*s*_ were assessed (Table 3). There was a positive relationship between *θ*_*mh*_ and BMC $\left({\hat{\beta }}_{\mathrm{\theta mh}}\;=\;20.94,\;p\;=\;0.001\right)$ and a positive relationship between *θ*_*s*_ and BMC $\left({\hat{\beta }}_{\mathrm{\theta s}}\;=\;27.77,\;p\;=\;0.001\right)$. There was a gender interaction with *θ*_*s*_ leading to an additional negative effect for boys on BMC $\left({\hat{\beta }}_{\mathrm{\theta s},\;\mathrm{boys}}\;=\;-36.03,\;p\;=\;0.006\right)$. By way of example, the fitted model predicts that for a girl a change in proportions of activity levels from (*π*_*s*_*π*_*l*_ *and π*_*mh*_)  =  (60*%*,  30*%*,  10*%*) to (65*%*,  27*%*,  8*%*) will lead to a change in BMC of $20.94\;*\;\log\left(\frac{0.08}{0.27}\right)\;+\;27.77\;*\log\left(\frac{0.65}{0.27}\right)-20.94*\log\left(\frac{0.1}{0.3}\right)-27.77*\log\left(\frac{0.6}{0.3}\right)=2.7g,$ whereas it will be −4.0 g for a boy. In a different example we observe a change in proportions of activity levels from (π_*s*_*,*π_*l*_ and π_*mh*_) = (70%, 22%, 8%) to (75%, 15%, 10%) will lead to a change in BMC of $20.94\;*\;\log\left(\frac{0.1}{0.15}\right)\;+\;27.77\;*\log\left(\frac{0.75}{0.15}\right)-20.94*\log\left(\frac{0.08}{0.22}\right)-27.77*\log\left(\frac{0.70}{0.22}\right)=25g$, whereas it will be 8.9 g for a boy.

**Table 3:** Effect of the physical activity on BMC at follow up

**Variable**	**Coefficient estimate**	**Standard error**	**P- value**
BMC _baseline_	0.44	0.03	<0.001
BA follow up	0.97	0.04	<0.001
Gender	35.20	9.50	<0.001
$\theta_{\mathrm{mh}}\;=\;\text{log}\left(\frac{\pi_{\mathrm{mh}}}{\pi_{l}}\right)$	20.94	6.58	0.001
$\theta_{s}\;=\;\text{log}\left(\frac{\pi_{s}}{\pi_{l}}\right)$	27.77	8.17	0.001
Gender # *θ*_*s*_	−36.03	13.11	0.006
Height _follow up_	−3.96	0.17	<0.001
Puberty _follow up_	29.99	3.52	<0.001
Gender # puberty	−16.95	5.14	0.001

Note: *π*_*mh*_*,π*_*s*_*, π*_*l *_represents the percentage of the total time spent in, moderate-high, sedentary and low intensity activity respectively. #: Interaction. The girls were chosen as the reference level for gender.

The relationship between BMD and the PA intensity levels represented by *θ*_*mh*_ and *θ*_*s*_ were assessed (Table 4). There was a positive relationship between *θ*_*mh*_ and BMD $\left({\hat{\beta }}_{\mathrm{\theta mh}}\;=\;0.009,\;p\;<\;0.001\right)$. There was no significant relationship between *θ*_*s*_ and BMD and no gender interaction.

**Table 4:** Effect of the physical activity on BMD at follow up

**Variable**	**Coefficient estimate**	**Standard error**	**P- value**
BMD _baseline_	0.966	0.02	<0.001
$\theta_{\mathrm{mh}}\;=\;\text{log}\left(\frac{\pi_{\mathrm{mh}}}{\pi_{l}}\right)$	0.009	0.001	<0.001
Height _follow up_	0.001	0.0002	<0.001
Log(weight _follow up_)	0.039	0.008	<0.001
Gender	0.017	0.005	0.001
Puberty _follow up_	0.016	0.002	<0.001
Gender # puberty^1^	−0.014	0.003	<0.001
Intercept	−0.150	0.018	<0.001

Note: *π*_*mh*_*,π*_*l *_represents the percentage of the total time spent in, moderate-high and low intensity activity respectively. #: Interaction. The girls were chosen as the reference level for gender.

The relationships between BA and the PA levels represented by *θ*_*mh*_ and *θ*_*s*_ were assessed (Table 5). There was a positive relationship between *θ*_*mh*_ and BA $\left({\hat{\beta }}_{\mathrm{\theta mh}}\;=\;22.18,\;p\;<\;0.001\right)$ but no significant relationship between *θ*_*s*_ and BA and further there was no significant gender interaction.

**Table 5:** Effect of the physical activity on BA at follow up

**Variable**	**Coefficient estimate**	**Standard error**	**P - value**
BA _baseline_	0.62	0.49	<0.0001
$\theta_{\mathrm{mh}}\;=\;\text{log}\left(\frac{\pi_{\mathrm{mh}}}{\pi_{l}}\right)$	22.18	4.20	<0.001
Height _follow up_	6.87	0.69	<0.001
Log(weight _follow up_)	285.20	18.12	<0.001
Puberty _follow up_	24.47	2.32	<0.001
Gender	−26.33	5.52	<0.001
Intercept	−1405.85	86.01	<0.001

Note: *π*_*mh*_*,π*_*l *_represents the percentage of the total time spent in, moderate-high and low intensity activity respectively. The girls were chosen as the reference level for gender.

The graphs in Figure 1 illustrate the impact on bone health represented by BMC, BMD and BA when a certain level of physical intensity was kept fixed at the mean value allowing an exchange of time spent in the two remaining intensity levels to occur. This exchange occurred between the time spent in the two remaining intensity levels at their 10th, 25th, 50th, 75th and 90th percentiles. The solid lines refer to the girls whereas the dotted lines refer to the boys.

In the first column sedentary intensity level is fixed at the mean value 64% and 62% of the total time in activity for girls and boys respectively. An exchange between low-level activity and moderate to high level activity can occur and when increasing the proportion of time in low-level activity the BMC, BMD and BA values will decrease for both genders. In the second column low intensity PA is fixed at the mean level 29% of the total time in activity for boys and girls, and an exchange between moderate to high and sedentary can occur. The bone traits increases as the proportion of time spent in moderate to high-level PA increases opposed to sedentary level PA. In the third column moderate to high level PA is fixed at the mean value 7% and 9% of the total time in activity for girls and boys respectively. An exchange between sedentary level activity and low-level activity reveals an increase in bone outcome when the proportion of time in sedentary level activity increases opposed to low-level activity.

## Discussion

A major reason for the interest in increasing bone health during growth is to prevent fractures due to osteoporosis later in life. This longitudinal study examined the relationship between the proportion of time spent at PA in different intensity levels and bone health represented by BMC, BMD, and BA accrual during two years, and showed a positive relationship of the log odds of moderate to high and low intensity activity and BMC, BMD and BA accruement over a two- year period.

We also found a significant relationship between the log odds of sedentary relative to low intensity activity and BMC as well as a significant gender interaction with an additional negative effect on BMC for boys. The changes in particular configurations between the three categories of PA revealed positive effects on bone traits when increasing the proportion of time in moderate to high-level activity opposed to sedentary and low-level activity behavior, but also a positive effect on bone traits when increasing the proportion of time in sedentary activity on behalf of low level activity. This rather surprising but interesting result may reflect that sedentary behavior is not necessarily negative for the bones compared to a general low activity level. However, this result was only found for BMC and not BMD and BA and should be confirmed in other studies.

The positive relationship between weight-bearing exercises, and bone health during growth has been well described [22]. However, this study provides additional information about the association between children’s habitual PA reported as the relationships between the proportion of time spent in different intensity levels of PA and the outcome variables BMC, BMD and BA accrual.

The strengths of the study included the population size, the large numbers of participants at follow up and the longitudinal design. There was an equal distribution between genders, and children attending sports schools and normal schools. The data collection included the gold standard method of measuring bone health parameters, DXA, and objective information on the children’s physical activity (PA) level. Assessment of PA by accelerometers provided valid information on the frequency, intensity and duration of PA. The accelerometer output correlated well with ground reaction force (GRF) which is pertinent to bone health [23]. The objective measures of accelerometers had the advantage that the children did not need to recall behaviors of physical activity, and therefore output did not rely on cognitive ability and recall bias.

Accelerometers have limitations as to the technical specifications and the participant’s willingness to wear the accelerometers. However, we succeeded in obtaining complete datasets on 81% of the participants. Another positive aspect of our study was that the children included in the analyses were measured with accelerometers at a mean of 6.1 days (4–11 days) and at a mean of 13 hours per day (11–14 hours per day). Trost *et al.* demonstrated that 4–5 days of monitoring were needed to obtain an intra class coefficient of 0.8 in children [24], which we exceeded. The analyses only addressed the effect of the relative distribution of PA among three levels. Although the total amount of activity may have an impact on the bone health, the data analyzed here are not amenable for this question.

Some other methodological aspects need to be mentioned. The collection of accelerometer data was performed at one-year follow-up. We had no knowledge of how this one period of measurements represented the child’s activity level over a longer time period, but we assumed that the measurements reflected the child’s activity level in general. This assumption was based on the knowledge that although PA and activity patterns varies from day to day as well as by season [25], children exhibit less day-to- day variability than adults [24]. PA measurements by accelerometers were obtained during the same yearly season for all children. Diet was not considered in this study. However, the children’s diet habits and variation in these may serve as a possible confounding factor when monitoring bone health.

Our findings of an association between the intensity levels of PA and bone traits corresponded well with the findings in a study by Tobias JH *et al.* (2007) in which the results presented were derived from a cross-sectional design [13]. In a previous study, Sardina *et al.* (2008) examined the relationship between intensity and duration of physical activity and composite indices of femoral neck strength and bone mineral content of the femoral neck, lumbar spine and total body. They concluded that vigorous activity measured by accelerometers emerged as the main PA predictor of femoral neck strength [26]. This corresponded well with our results, although we measured TBLH BMC, BMD, and BA, and not indices of bone strength. We found that greater discrepancies in activity level are necessary to disclose the effects of physical activity on bone accretion.

Several other studies have presented cross-sectional data on the positive relationship between habitual levels of physical activity at different intensity and bone mass, in particular moderate to high intensity PA [12,13,27]. Our study provides longitudinal data from a large two-year follow up study.

Habits of physical activity can be traced from childhood to adolescence and adulthood [28,29], and habits developed in early life may persist into adulthood. It has been suggested that children become less physically active and spend more time in sedentary activity as they age [30]. However, this suggestion has been questioned in other studies [31,32]. Our graphic presentation of the data emphasizes the importance of spending a higher proportion of the total time in higher activity levels to maintain a beneficial bone mineral accrual during a two-year observational time. Our results suggest that small changes in PA behavior towards more moderate to high level activity opposed to low and sedentary intensity levels are sufficient to achieve these beneficial effects on bone traits.

In recent years, osteoporosis has been recognized as a growing problem in adults and the elderly [1,2]. When planning prevention strategies towards osteoporosis, all of the factors that affect bone health including the size of PBM should be considered. Bone growth during childhood and adolescence is important for reaching optimal PBM [7]. Thus it is crucial to optimize these modifiable factors with regard to bone development in childhood. Although up to 60-80% of the variance in PBM is accounted for by genetic factors, the remaining 20-40% are influenced by lifestyle factors such as physical activity [7,33].

Focus on habitual physical activity during childhood is of major importance to consider when planning preventive strategies towards osteoporosis. Also in relation to bone health it is important to change children’s lifestyle from mainly inactivity to higher activity levels.

## Conclusion

A positive association was observed between physical activity and bone mineral accretion in childhood. In particular moderate to high intensity activity had a positive influence on the bone health. The data suggest that changes in the distribution of time spent in different activity intensity levels will influence the bone mineral accruement during a two year period with moderate to high intensity activity having a positive effect on bone outcome, whereas an increase in low-level and sedentary activity opposed to moderate to high-level activity will have a negative impact on bone health in childhood. This knowledge has public health as well as clinical relevance. Further research should focus on detecting the threshold of the beneficial effects of physical activity on bone health.

## Competing interests

All authors state that they have no competing interest.

## Authors’ contributions

Study design: MH, CM, NW, SH, HK, NC, AJS. Study conduct: MH, HK, NC. Data collection: MH, HK, NC. Data interpretation: MH, CM, NW, RH and NC. Drafting manuscript: MH, CM, and NW. Revising content: MH, CM, NW, SH, HK, NC, AJS, RH. Approving the final version of the manuscript: MH, CM, NW, SH, HK, NC, AJS, RH. MH takes responsibility for the integrity of the data analyses.

## Pre-publication history

The pre-publication history for this paper can be accessed here:

http://www.biomedcentral.com/1471-2431/13/32/prepub
